# Zero‐fluoroscopy catheter ablation of premature ventricular contractions at left coronary cusp near left main coronary artery

**DOI:** 10.1002/ccr3.3035

**Published:** 2020-06-22

**Authors:** Amato Santoro, Claudia Baiocchi, Flavio D’Ascenzi, Sergio Mondillo, Serafina Valente

**Affiliations:** ^1^ Cardiology Department Azienda Ospedaliera Universitaria Senese Viale Bracci 1, Siena Italy

**Keywords:** catheter ablation, premature ventricular contraction, zero‐fluoroscopy

## Abstract

The left coronary cusp is the commonest site of origin for coronary cusp PVC. Catheter ablation without fluoroscopy is highly effective, feasible, and safe but it could be related to risks because of proximity to the coronary arteries. The use of ICE integration allowed an improvement in the safety and efficiency of these procedures.

## Graphical abstract

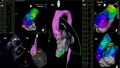

## 1 INTRODUCTION

Premature ventricular contractions (PVCs) are ectopic heartbeats caused by early myocardial depolarization. Baman et al^1^ suggested that a PVC burden >24% can contribute to cardiomyopathy and heart failure. In other studies, the threshold burden of PVC for reduced left ventricular (LV) ejection fraction (EF) was ≥10%.^2^ Catheter ablation (CA) is an effective and relatively safe treatment for patients with symptomatic frequent PVCs.^3^ The site of origin of the earliest activation is the target of ablation for focal PVCs.^5^

## 2 CASE PRESENTATION

**Figure 1:**
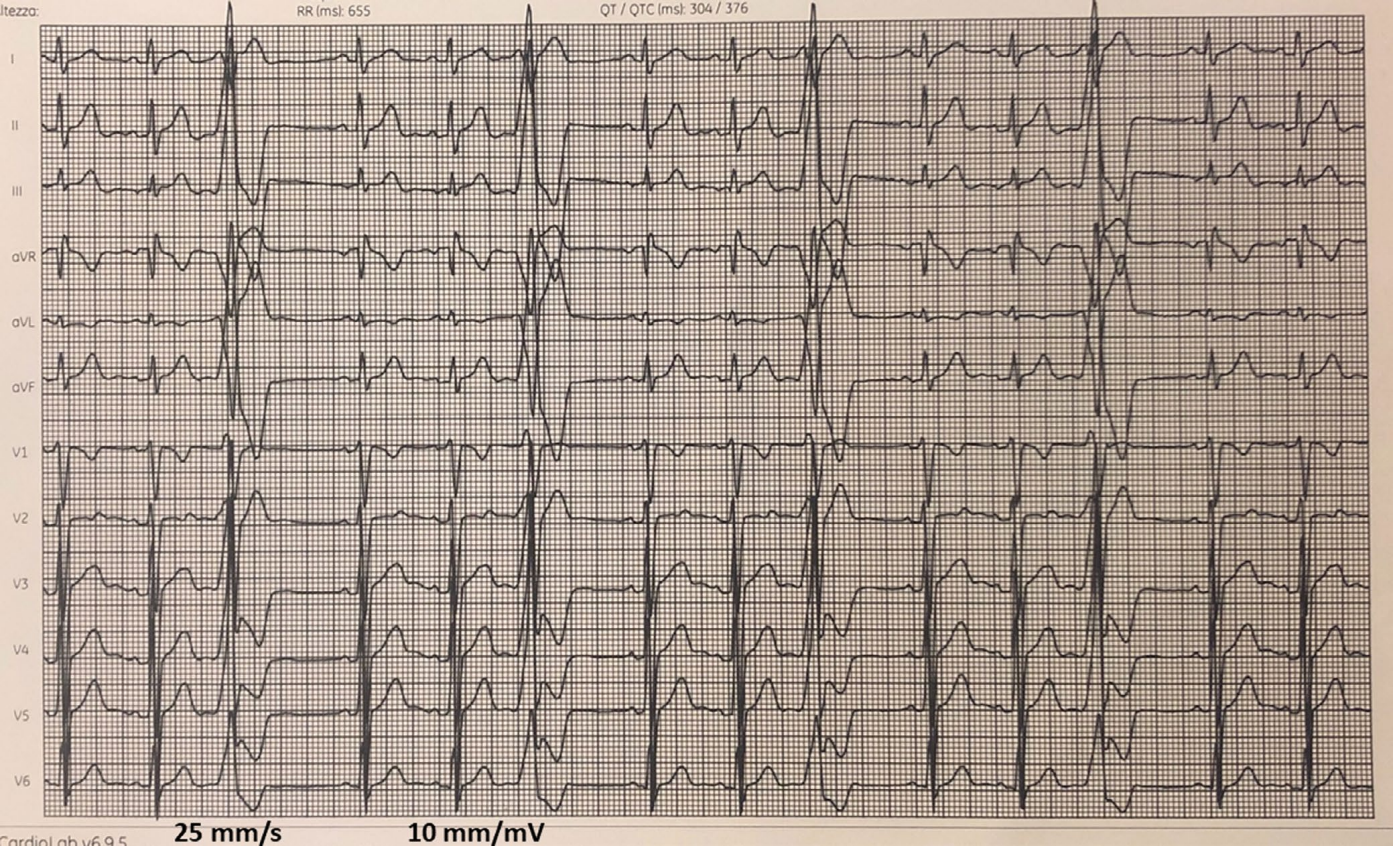
ECG at admission in Hospital

**Figure 2:**
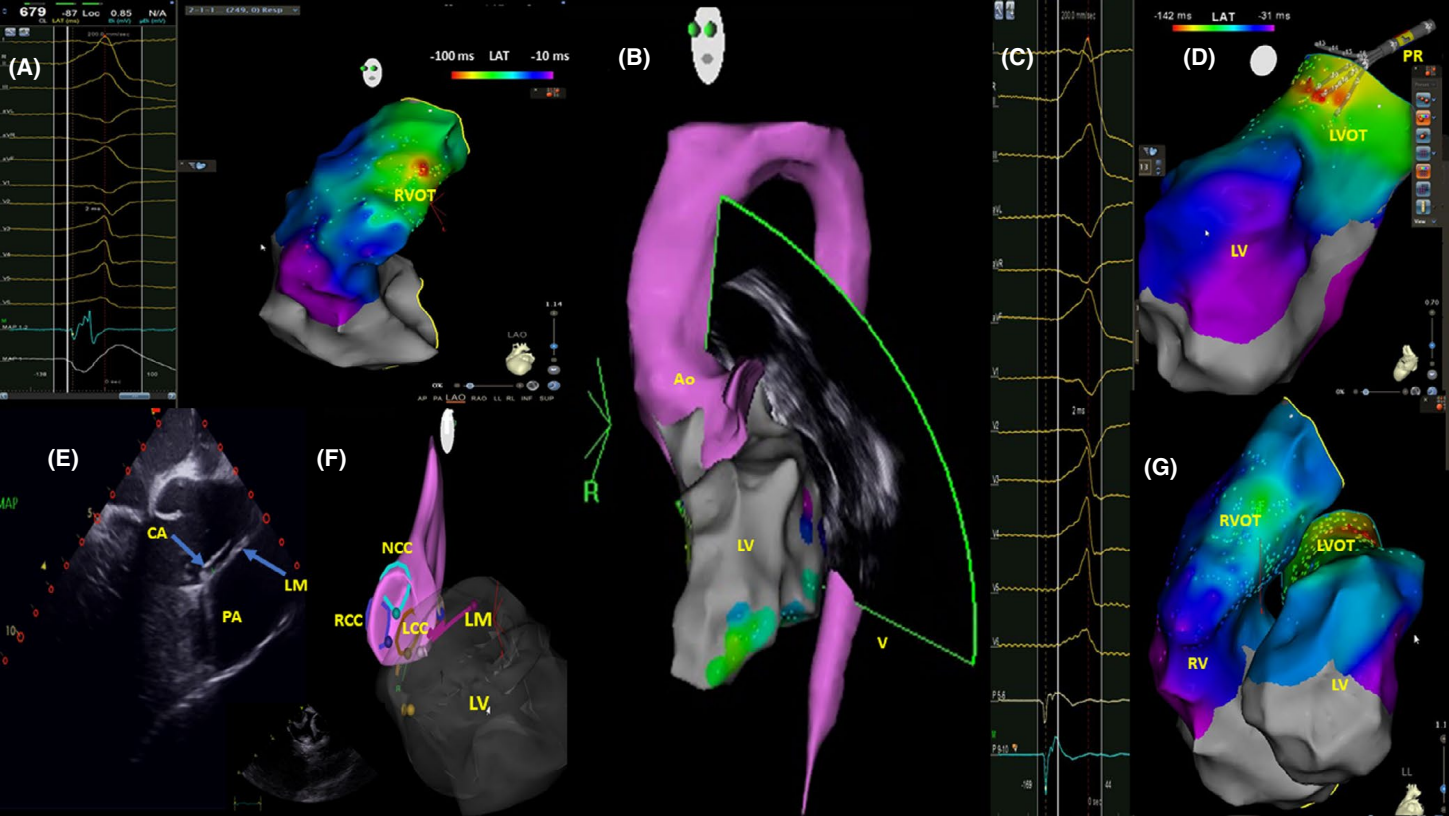
A, EGM and LAT during RVOT mapping. B, The anatomical Carto‐map that showing left ventricle and aorta. C, EGM stored using pentaRay catheter during LVOT mapping. D, LAT during LVOT mapping. E, 2D intracardiac echocardiography. The tip of the ablation catheter was positioned near left main coronary artery. F, ICE map with LCC and LM, RCC, NCC, and LV merged with anatomical map of Aorta and left ventricle; G, LAT of left and right ventricles; the earliest point of activation shown in the column was registered in LVOT, at left coronary cusp, near left main coronary artery. Ao, aorta; CA, tip of ablation catheter; EGM, electrograms; ICE, intracardiac echocardiography; LAT, local activation time; LCC, left coronary cusp; LM, left main coronary artery; LV, left ventricle; LVOT, left ventricle outflow tract; NCC, no coronary cusp; RCC, right coronary cusp; RVOT, right ventricle outflow tract

**Figure 3:**
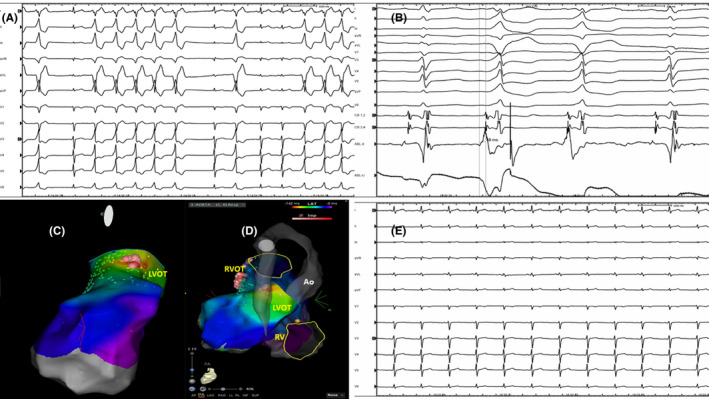
A, ECG at the begin of catheter ablation; B, intracardiac electrogram at ablation point; early activation with a sharp unipolar QS electrogram at successful ablation site. C, LV EAM with Visitag ablation's points on early activation EGM; strict Visitag stability settings were applied (1 mm, 5 s) during a single RF application; the different colors of tags were related to impedance drop during RF. The RF application is continued until the color of Visitag changes from white to red; the quite broad Visitags were related to minimal movements of ablation catheter and to minimal drops impedance; these tags showed a white or lightly red tag; the red tag corresponded to high impedance drop associated to real stability of ablation catheter. D, LAT and tags on unsuccessful early RVOT EGM and on successful earliest LCC EGM. E, final ECG. Ao, aorta; LVOT, left ventricle outflow tract; RV, right ventricle; RVOT, right ventricle outflow tract

A 24‐year‐old man arrived in our hospital complaining of palpitations, dyspnea, asthenia, and fatigue. A 12‐lead electrocardiogram (ECG, Figure 1) showed sinus rhythm with a high PVCs burden (rs in V1, RS transition in V3, rs in DI, and monophasic R in II, III, and aVF) and numerous episodes of nonsustained ventricular tachycardia (NSVT). Transthoracic echocardiography showed a moderately reduced systolic function (EF: 40%) and increased end‐diastolic diameter (70 mm) of the LV. Cardiac nuclear magnetic resonance imaging showed a structurally normal heart without fibrosis at delayed gadolinium enhancement imaging. The patient was treated with Metoprolol 25 mg and Flecainide 100 mg both twice daily without a reduction of PVC burden. The 24‐hour Holter monitor ECG recorded 45 000 monomorphic left bundle branch block‐type PVCs with numerous episodes of NSVT. The PVC burden was 45%. The patient was considered eligible for CA^4^ which was performed by using a proprietary navigation system for electroanatomic mapping (EAM) and ablation (Biosense Webster CARTO 3 System), with multipolar PentaRay advanced diagnostic 20 pole‐catheter, Cartosound advanced three‐dimensional (3D) mapping module, and Thermocool SF bidirectional therapeutic ablation catheter; Biosense Webster is a division of Johnson and Johnson, Inc. All medical and nursing staff have not worn lead aprons as reported in a previous observational study of our division of electrophysiology.^6^ The patient had a small lead apron over the genital area. After attaining 2 venous femoral vascular echo‐guided accesses, an anatomical 3D reconstruction and EAM of the right ventricular outflow tract (RVOT) was created (Figure 2A). Briefly sequential two‐dimensional (2D) intracardiac echocardiography (ICE) contours were acquired and used to create a 3D map of the RVOT ventricles and pulmonary cusps using ICE with the Cartosound module. There was a septal RVOT signal with an early activation of −10 ms and an unipolar QS signal. Thus, single 20‐W radiofrequency (RF) pulse was delivered to this point with the Thermocool SF catheter, but was not successful (Figure 2A). An echo‐guided right common femoral artery access was obtained. A 3D reconstruction of the coronary ostia and LV was performed with ICE. A local activation time (LAT) map of the left ventricular outflow tract (LVOT) and the aorta was produced with the PentaRay catheter (Figure 2B‐D). The earliest bipolar activation (−48 ms) was recorded at 4 mm below the ostium of the left main coronary artery when the ICE was merged with the EAM (Figure 2E‐G). Figure 3A,B respectively shows ECG trace before RF. There was a sharp, unipolar QS at the point of earliest activation (−48ms; Figure 3B) on the ablation catheter. A 30‐s 20‐W RF pulse was delivered to this point with the ablation catheter, and there was a transient suppression of PVCs after a single 9‐beat episode of NSVT that was attributed to irritation during RF. However, PVCs reoccurred at 1 minute after RF application, and a second 20‐s 30‐W pulse was delivered, followed by further suppression of PVCs. Another 60‐s 30‐W RF pulse was delivered, and the impedance was then lowered from 150 ohms (Ω) to 135 Ω. After this, the patient has maintained sinus rhythm without PVCs (Figure 3C‐E). The entire procedure was fluoroless. Fluoroscopy time during CA was 0 second, and coronary angiography was not performed before or after CA. PVCs were absent from the 24‐hour Holter ECG at the 1‐month follow‐up, and after 6 months, the patient underwent cycloergometric maximal exercise testing without ST‐T ECG changes and with no incidence of PVCs. At transthoracic echocardiography, the systolic function was normal (EF:65%), and the end‐diastolic LV diameter was reduced to 55 mm. A stress‐echocardiography, performed after 6 months, was negative for ischemia and ECG signs. The patient's eligibility for competitive sport was restored.

## 3 DISCUSSION

The presence of an R wave in V_1_ and V_2_ and R/S transition in leads V_1_ or V_2_ are characteristic of an LVOT origin. For LVOT PVC, the absence of an S wave in leads V5 or V6 suggests a supravalvular origin.^7^ The origin from the aortic cusp is also suggested by a longer duration and greater amplitude of the R wave in leads V_1_ or V_2._ Furthermore, a greater R wave amplitude in the inferior leads is observed in the left coronary cusp PVC (LCC). As the origin moves from the right coronary cusp (RCC) to the LCC, an S wave appears in I, the lead III/II ratio becomes greater, the precordial transitional zone moves clockwise. On ECG, right bundle branch and QRS morphology with a right inferior axis is observed in PVC with an LCC origin.^8^ Instead, the absence of an R wave in V_1_ or rS morphology in lead V_1_ and V_2_ (as shown in the ECG of this case) and R/S wave transition between leads V_3_ and V_4_ predict the earliest ventricular activation located at the posterior part of superior septal RVOT.^7^ However, in some cases, PVC with a QRS morphology characteristic of the RVOT origin could have an LVOT origin. It is very difficult to separate the coronary cusp focus from RVOT PVC, because they are so close to each other.^8^ The QRS morphology of outflow PVC can be affected by several factors such as lead position, cardiac anatomy, cardiac rotation, ventricular hypertrophy, breast size, body physique, gender, chest wall deformities, and conduction across the ventricular outflow tract septum.^9^ Differentiation between left vs right site of origin especially in cases with early activation site (EAS) located in the septal RVOT, in which the sensitivity and specificity of ECG criteria, has been shown to be lower than described.^10^ Despite the absence of epicardial connections, subendocardial fibers connect both out‐flow tracts across the infundibular septum. At the level of the LCC, this connection has been observed to take place within 1 cm below the pulmonary valve (PV), explaining the overlap observed in EAS‐PV distance between PVCs arising from LCC (or proximity) and those with RVOT origin.^10^ The left coronary cusp is the most common site of origin for coronary cusp PVC. Catheter ablation is highly effective in the management of patients with symptoms, LV dysfunction, or very frequent PVCs, but also carries risks because of proximity to the coronary arteries. Usually, the coronary angiography is recommended before and during CA to make sure there is a safe distance from the coronary ostia. The ability to produce 3D maps by using ICE merged with EAM permits thorough examination of the anatomical structures of the heart while avoiding coronary angiography and limiting risk.^11^ The coronary angiography presents risks, including allergic reaction or renal injury from contrast use, vascular injury from a second arterial access, additional radiation exposure, and rarely, air embolus or coronary dissection as reported in previous studies.^5^ Thus, the combination of EAM and ICE provided a more versatile and precise tool compared to the flat two‐dimensional fluoroscopic views. ICE is also helpful in confirming adequate tissue apposition of the ablating catheter, monitoring the pericardium, as well as promptly recognizing iatrogenic pericardial effusion. Furthermore, radiation exposure is associated with acute and subacute skin injury, malignancies, cataracts, thyroid dysfunction, and other diseases.^11^ Because of these potential risks and the linear relationship between radiation dose and increased risk of future malignancy (ie, no dose of radiation is considered safe), the underlying principle of radiation exposure states that the radiation dose must be "as low as reasonably achievable" (ALARA).^12^, ^13^ The patient of this case underwent RF ablation of PVCs without coronary angiography using a zero‐fluoroscopy approach. After 6 months of follow‐up, our patient had normal systolic and diastolic ventricular function and there were no complications. Most operators have suggested a cutoff of 10 mm from the coronary ostium as a safe distance for ablation, although others have suggested that ablation beyond 5 mm may be considered safe.^2^ In this case, the distance was 4 mm and the CA was performed without complications. We find that with careful use of ICE and EAM, coronary angiography can be avoided.

## CONFLICT OF INTEREST

None declared.

## AUTHOR CONTRIBUTIONS

AS: performed catheter ablation and wrote this paper. CB: performed catheter ablation as second operator. FD’A and SM: performed the ambulatory follow‐up of the patient. SV: is the head‐director of Division of Cardiology.
